# Stepped care targeting psychological distress in head and neck and lung cancer patients: a randomized clinical trial

**DOI:** 10.1186/1471-2407-12-173

**Published:** 2012-05-10

**Authors:** Anne-Marie H Krebber, C René Leemans, Remco de Bree, Annemieke van Straten, Filip Smit, Egbert F Smit, Annemarie Becker, Guus M Eeckhout, Aartjan TF Beekman, Pim Cuijpers, Irma M Verdonck-de Leeuw

**Affiliations:** 1 Department of Otolaryngology/Head and Neck Surgery, VU University Medical Center, Amsterdam, the Netherlands; 2 Department of Clinical Psychology, VU University, Amsterdam, the Netherlands; 3 Department of Pulmonary Diseases, VU University Medical Center, Amsterdam, the Netherlands; 4 Department of Psychiatry, VU University Medical Center, Amsterdam, the Netherlands; 5 Department of Otolaryngology / Head and Neck Surgery, VU University Medical Center, PO BOX 7057, 1007 MB Amsterdam, The Netherlands; 6 Trimbos Institute, Netherlands Institute of Mental Health and Addiction, , Utrecht, The Netherlands; 7 Department of Epidemiology and Biostatistics, EMGO Institute for Health and Care Research, VU University Medical Center, Amsterdam, The Netherlands

**Keywords:** Cancer, Distress, Anxiety, Depression, Psychosocial care, Stepped care, Screening, Quality of life, Efficacy

## Abstract

**Background:**

Psychological distress is common in cancer survivors. Although there is some evidence on effectiveness of psychosocial care in distressed cancer patients, referral rate is low. Lack of adequate screening instruments in oncology settings and insufficient availability of traditional models of psychosocial care are the main barriers. A stepped care approach has the potential to improve the efficiency of psychosocial care. The aim of the study described herein is to evaluate efficacy of a stepped care strategy targeting psychological distress in cancer survivors.

**Methods/design:**

The study is designed as a randomized clinical trial with 2 treatment arms: a stepped care intervention programme versus care as usual. Patients treated for head and neck cancer (HNC) or lung cancer (LC) are screened for distress using OncoQuest, a computerized touchscreen system. After stratification for tumour (HNC vs. LC) and stage (stage I/II vs. III/IV), 176 distressed patients are randomly assigned to the intervention or control group. Patients in the intervention group will follow a stepped care model with 4 evidence based steps: 1. Watchful waiting, 2. Guided self-help via Internet or a booklet, 3. Problem Solving Treatment administered by a specialized nurse, and 4. Specialized psychological intervention or antidepressant medication. In the control group, patients receive care as usual which most often is a single interview or referral to specialized intervention. Primary outcome is the Hospital Anxiety and Depression Scale (HADS). Secondary outcome measures are a clinical level of depression or anxiety (CIDI), quality of life (EQ-5D, EORTC QLQ-C30, QLQ-HN35, QLQ-LC13), patient satisfaction with care (EORTC QLQ-PATSAT), and costs (health care utilization and work loss (TIC-P and PRODISQ modules)). Outcomes are evaluated before and after intervention and at 3, 6, 9 and 12 months after intervention.

**Discussion:**

Stepped care is a system of delivering and monitoring treatments, such that effective, yet least resource-intensive, treatment is delivered to patients first. The main aim of a stepped care approach is to simplify the patient pathway, provide access to more patients and to improve patient well-being and cost reduction by directing, where appropriate, patients to low cost (self-)management before high cost specialist services.

**Trial registration:**

NTR1868

## Background

Every year more than 14,000 patients are diagnosed with cancer, of whom 11,470 with lung cancer (LC) and 2,870 with head and neck cancer (HNC) in the Netherlands [1]. Five-year survival rates are estimated at 13% in LC and 50% in HNC. Approximately 80% of the LC patients are male compared to 65% of the HNC patients. Patients often have to deal with devastating side effects of initial treatment (surgery, radiotherapy, and/or chemotherapy), such as pain, fatigue, and respiratory, speech and swallowing problems, negatively affecting health-related quality of life and associated with increased levels of psychological distress. Co-morbid anxiety or depression is present in 20-30% of LC and HNC patients [2-4]. During the first year after treatment there is a gradual improvement of psychological functioning [5,6] but many patients continue to suffer from or develop anxiety or depression [2,7-9].

Because of the overwhelming evidence of psychological distress in LC and HNC patients, intervention is recommended in national guidelines. Some recent reviews have shown evidence on efficacy of psychosocial intervention in cancer patients in general [10,11]. Others question evidence mainly because randomized trials are scarce [12,13] and because most studies included all patients even those without symptoms of depression and anxiety. Furthermore, it appears that most intervention studies are applied in patients with breast cancer. Patients with less prevalent tumours such as HNC or poor survival rates as in LC are often not involved, while LC and HNC patients are among the most distressed patients compared to cancer patients in general [14].

In clinical practice at present, many cancer patients who report high levels of psychological distress are not taking advantage of psychosocial care [14,15]. Barriers to admission to adequate psychosocial care are a lack of adequate screening of anxiety and depression in the often very busy oncology settings, reluctance by patients to be referred because of the already long treatment period, and that traditional models of the delivery of psychosocial care cannot meet current demand. Other forms of delivery, such as brief therapies, group treatments and self-help, and a stepped care approach may provide useful alternatives. Studies regarding cost-effectiveness of psychosocial intervention in cancer patients are scarce [16].

Stepped care algorithms are based on clinically proven, best-practice pathways to care over a series of steps, while taking into account patients’ preference [17]. The steps involve watchful waiting, guided self-help and other brief therapies, followed by more intensive psychological interventions or medication. In stepped care, more intensive treatments are generally reserved for people who do not benefit from simpler first-line treatments, or for those who can be accurately predicted not to benefit from such treatments. The results of treatments and decisions about treatment provision are monitored systematically, and changes are made (‘stepping up’) if current treatments are not achieving a significant health gain [10]. Stepped care models have been developed for several health problems, including smoking, back pain, alcohol treatment, migraine, anxiety, eating disorders, methadone maintenance, and depression [10].

The main goal of the proposed study is to assess efficacy of a stepped care strategy compared to care as usual in patients with psychological distress after treatment for LC or HNC to improve psychological distress and thereby quality of life.

## Methods/Design

### Design

In this prospective randomized controlled trial in two parallel groups, patients are recruited by screening all LC and HNC patients, who visit the Department of Pulmonary Diseases or the Department of Otolaryngology and Head and Neck Surgery of the VU University Medical Center in Amsterdam, the Netherlands, for follow-up consultation at least one month after treatment, for distress using a computerized touch screen data collection system (OncoQuest) or by telephone using the Hospital Anxiety and Depression Scale (HADS). All patients who fulfil the in- and exclusion criteria are asked to participate. After stratification for tumour site (LC vs. HNC) and stage (stage I-II versus III-IV), 176 participating patients are randomly assigned to the intervention or control group. In order to assess efficacy, assessment before and after intervention takes place and at 3, 6, 9, and 12 months follow up.

### Study sample

Inclusion criteria are: treatment for UICC stage I-IV lung or head and neck carcinoma: ICD-10 C00-C14 (lip, oral cavity and pharynx), C32 (larynx), C33 (trachea), C34 (lung); psychological distress or possible or probable cases of depression or anxiety as assessed by the Hospital Anxiety and Depression Scale (HADS; HADS-D > 7 or HADS-A > 7 or HADS-total > 14).

Exclusion criteria are: other (neurological) diseases causing cognitive dysfunction; no motivation to undergo psychological therapy; current treatment for a depressive or anxiety disorder; end of treatment for a psychiatric disorder less than two months ago; high suicide risk; psychotic and/or manic signs; too little knowledge of the Dutch language to fill out the questionnaires.

### Randomization

Randomization is conducted centrally by an independent statistician, in blocks of two, stratified for tumour site (LC vs. HNC) and stage (stage I-II vs. III-IV), because these variables have prognostic relevance and need to be distributed evenly across both conditions.

### Intervention

Patients in the experimental study arm enter a stepped care programme including 1) watchful waiting, 2) guided self-help via Internet or a booklet, 3) face-to-face problem solving treatment, 4) specialised psychological interventions such as cognitive behavioural therapy and/or antidepressant medication.

The basic proposition of stepped care is that all patients are offered the same low intensity (evidence-based) treatment as a first step. Only those patients, who do not recover, step up to a more intensive treatment. The HADS score is used to determine stepped-up levels of care. Stepping up to the next treatment level is indicated when a participant’s HADS-A or HADS-D score exceeds 7. The care coordinator controls the process, monitors the symptoms, and makes sure the patient steps up if necessary.

The stepped care programme in the present project includes the following four steps.

#### Step 1: Watchful waiting

In the first step it is agreed on not to start intervention yet, but to wait for further development of symptoms. Because part of the patients recovers spontaneously [18] ‘watchful waiting’ is included in the multidisciplinary guideline on depression as first treatment step. Duration of the watchful waiting period in the present project including cancer patients is set on 2 weeks.

#### Step 2: Guided self-help via Internet or a booklet

If there is no spontaneous recovery after 2 weeks, the care coordinator contacts the patient for one counselling session in which the self-help programme is introduced. As intervention the existing programme “Allesondercontrole” or the web based programme “Allesondercontrole” (http://allesondercontrole.psy.vu.nl) is used. “Allesondercontrole” is a brief intervention for problem-solving based on self-examination. “Allesondercontrole” is already available. The website is currently only used for research purposes and both international and national research has shown that this intervention is effective in depression and anxiety [19]. The intervention is based on problem-solving therapy, which has been proven to be effective in several randomized controlled studies [20], also when delivered via the Internet. A recent meta-analysis by our group found that the effects of Internet-based treatments of depression and anxiety disorders are as large as those of face-to-face treatments [21]. The intervention “Allesondercontrole” takes 5 weeks. In that period respondents describe what they think is important in their lives, make a list of their problems and concerns, and divide these into three categories: unimportant problems (problems which are not related to what is important in their life), important and amenable problems (these are solved through a six-step procedure of problem-solving), and important but unsolvable problems (such as having a serious disease like cancer); for each of the amenable problems the respondent makes a plan). Trained coaches guide the patients through this process. The coaching consists of brief, weekly contacts by email or by telephone, which takes about 10 to 15 minutes per week. The total coaching time is 1 to 1.5 hours per patient (estimation based on our previous trial). Coaching is not aimed at developing a patient-therapist relation but is only meant to give support in working through the self-help method.

#### Step 3: Face-to-face problem solving treatment

When the patient has not recovered from the guided self-help programme, a nurse from the department of Psychiatry offers a brief intervention: Problem Solving Treatment (PST). Earlier studies revealed that PST can be delivered by psychologists as well as nurses [22-24]. PST identifies problems that interfere with everyday functioning and that contribute to depression and anxiety. The treatment provides compensatory strategies that are designed to bypass the person's cognitive limitations and to improve adaptive functioning. PST comprises a short 6-session protocolled intervention. The first session takes 1 hour, the other sessions 45 minutes. PST is an evidence-based intervention for major depression and for psychological distress characterised by symptoms of depression and anxiety [22,23,25-28].

#### Step 4: Specialised psychological interventions such as cognitive behavioural therapy and/or antidepressant medication

**Figure 1:**
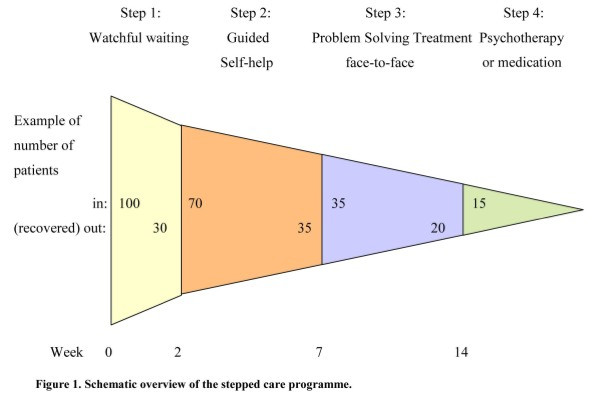
Overview of the stepped care programme.

In case all previous steps have not induced recovery, the patient chooses in close cooperation with the care coordinator between medication and psychotherapy. To ease this decision, the patient is offered the patient information letter of the Dutch College of General Practitioners on antidepressant medication. (A) If the patient chooses medication, the care coordinator contacts the patients’ physician who prescribes antidepressant medication and monitors outcomes. (B) If the patient chooses psychotherapy, the patient is referred to a psychologist or a psychiatrist. The stepped care programme is illustrated in Figure 1.

#### Care as usual

Control group patients receive care as usual, which often means a single interview by a nurse or specialised intervention delivered by a social worker, psychologist or psychiatrist. In the context of the health economic evaluation, health care uptake is closely monitored, thus allowing for detailed post-hoc description of what usual care entailed exactly.

### Outcome assessment

#### Main outcome measure

The primary outcome measure is the Hospital Anxiety and Depression Scale (HADS). The Hospital Anxiety and Depression Scale (HADS) is a 14-item self-assessment scale for measuring symptoms of anxiety and depression and has been specifically designed for use in the medical ill. This scale has been proven to have adequate psychometrical properties and the total HADS score has been recommended for routine monitoring of psychological distress in cancer patients [29-32].

#### Secondary outcomes

Secondary outcome measures are health related quality of life questionnaires (EORTC QLQ-C30), EORTC QLQ-HN35, EORTC QLQ-LC13), general patient satisfaction (EORTC QLQ-PATSAT), and economic evaluation.

The EORTC QLQ-C30 is a tumour-specific, patient-based questionnaire. The questionnaire includes a global HRQOL scale (2 items) and comprises 5 functional scales: physical functioning (5 items), role functioning (2 items), emotional functioning (4 items), cognitive functioning (2 items) and social functioning (2 items). There are three symptom scales (fatigue (3 items), nausea, vomiting (2 items) and pain (2 items) and 6 single items relating to dyspnoea, insomnia, loss of appetite, constipation, diarrhoea and financial difficulties [33].

The EORTC QLQ-LC13 module covers specific issues on lung cancer. The questionnaire comprises 12 symptom scales: a 3 item scale on dyspnoea, and 1 item scales on coughing, haemoptysis, sore mouth, dysphagia, peripheral neuropathy, alopecia, pain in chest, pain in arm or shoulder, pain in other parts [33].

The EORTC QLQ-HN35 module covers specific issues on head and neck cancer. It has been used previously in studies. The questionnaire comprises 7 subscales: pain (4 items), swallowing (5 items), senses (2 items), speech (3 items), social eating (4 items), social contact (5 items) and sexuality (2 items). There are 10 single items covering problems with teeth, dry mouth, sticky saliva, cough, opening the mouth wide, weight loss, weight gain, use of nutritional supplements, feeding tubes, and painkillers [33,34].

The EORTC QLQ-PATSAT32 module is a patient satisfaction with care measure. The questionnaire comprises 4 scales on interpersonal skills (3 items), technical skills (3 items), information provision (3 items), and availability (2 items) of doctors, the same 4 scales regarding nurses, 1 scale on other hospital personnel kindness and helpfulness, and information giving (3 items), 1 scale on waiting time (2 items), 1 scale on access (2 items), and 3 single items on exchange of information, comfort/cleanness, and general satisfaction [35].

The economic evaluation will be conducted as a cost-utility analysis were outcomes are (changes in) health-related quality of life at patient level. Health-related quality of life is assessed with the EQ-5D [36,37] at baseline and 12 months follow-up. Direct medical and direct non-medical cost data are collected with the TIC-P [38], a widely used health service receipt interview in economic evaluations. Unit resource use (GP visits, hospital days, etc.) will be multiplied by their appropriate integral cost prices [39]. Indirect non-medical cost data related to production losses through work loss days and work cutback days will be sampled with the appropriate PRODISQ modules [40].

#### Sociodemographic and medical data

Next to the outcome measures, a case record form is developed including sociodemography (age, gender, social economic status), cancer and cancer treatment (TNM and ICD-10 classification, documentation of surgery and (chemo)radiation), and co-morbidity (Adult Comorbidity Evaluation 27 (ACE-27) test). The ACE-27 was designed specifically for cancer patients and classifies patients into 1 of 4 grades of comorbidity (none, mild, moderate, severe) [41].

#### Diagnostic evaluation

The presence of a major depression or an anxiety disorder is assessed according to the Composite International Diagnostic Interview (CIDI). The CIDI is a comprehensive, fully structured interview designed to be used by trained lay interviewers for the assessment of mental disorders according to the definitions and criteria of ICD-10 and DSM-IV. The diagnostic section of the interview is based on the World Health Organization's Composite International Diagnostic Interview [41-43].

### Statistical analyses

Descriptive statistics will be generated for the range of outcome variables, in particular to gauge whether randomization resulted in a balanced distribution of patients characteristics across the experimental conditions.

Repeated measures ANOVA will be used to determine the efficacy of intervention for continuous outcomes such as changes in HADS depression/anxiety symptom severity. Longitudinal changes over time in these variables will also be evaluation over all time points simultaneously using generalized estimating equations (GEEs). Analyses will be conducted in agreement with the intention-to-treat principle.

The economic data will be collected at baseline and follow-up and conducted as a cost utility analysis that is with health-related quality of life as the clinical endpoint. For the economic evaluation use will be made of the pertinent guidelines [39,44-46]. In other words, analyses will be conducted in agreement with the intention-to-treat principle; the societal perspective will be taken encompassing intervention costs, direct non-medical costs and indirect costs. Production losses will be economically valued using the friction cost method [47]. The time horizon will be set at one year, and therefore we will neither discount costs nor effects. Costs and effects will be analysed simultaneously, incremental cost-effectiveness ratios (ICERs) will be calculated and placed within 95% confidence intervals, 2,500 bootstrap replications of the ICERs will be projected on a cost-effectiveness plane, ICER acceptability curves will be plotted against different willingness-to-pay ceilings [48], and sensitivity analysis will be conducted as a matter of course focussing on uncertainty in the main cost-drivers. This will be done for the costs per QALY gained in a cost utility analysis.

### Sample size calculation

To demonstrate an effect size of 0.50 (based on a meta-analysis on psychological treatment in mild depression), 66 patients are needed in each group (power 80%, significance level 5%) [49]. Taking into account a dropout of 25%, in total 176 patients will be included. With an annual intake of 450 LC and HNC patients, 30% having psychological distress, and 50% willing to cooperate, and an inclusion period of 2.5 years, feasibility of the study is guaranteed.

## Discussion

There is a rising need towards screening for physical and psychosocial problems and the need for supportive care in routine clinical practice through patient-reported outcomes (PRO’s) [50-54]. The use of PRO’s has proven to facilitate communication concerning quality of life between patients and health care professionals [55-57]. Evidence that this approach may influence patient outcome or improve quality of life is scarce. Luckett and colleagues [54] recommend additional efforts to strengthen the effects of screening, such as using more tumour specific (instead of generic) PRO’s, improving the interpretability of feedback for both medical staff and patients, and training patients in self-efficacy. Organising supportive care according to the chronic care model [58] and providing evidence based supportive care can also improve disease management in cancer patients.

Disease management refers to a system of coordinated comprehensive care along the continuum of the disease across health care delivery systems, with a specific focus on self-management. Other forms of providing supportive care comprise integrated care, transmural care, collaborative care, case management, and stepped care. In oncological settings, recent projects as “Supporting transmural oncological care” [59] and “Integrated care” [60] revealed that supportive care coordination improves supportive care delivery in cancer patients. A review on professional patient navigation in head and neck cancer patients showed that the presence of a professional care navigator leads to higher patient satisfaction, shorter duration of hospitalization, fewer cancer-related problems, better emotional quality of life, and patient empowerment [61].

At present, in VU University Medical Center in Amsterdam, The Netherlands, efficient structured monitoring of quality of life by a touchscreen computer-based data collection system “OncoQuest” is implemented in routine clinical practice [15,62]. Patients can independently fill in the EORTC QLQ-C30, and tumour specific modules, and the Hospital Anxiety and Depression Scale (HADS) on a touchscreen. It takes on average 9 minutes to complete the questionnaires. Data are processed in real-time and care providers can watch the results by clear charts (the well-being profile) on a computer in their consulting rooms and, if indicated, set up a custom-made supportive care plan. Nurses are trained as care navigators to arrange the supportive care according to the disease management principles.

From an economic perspective and in an age of increasing numbers of cancer survivors and increasing shortages of health care personnel, it is relevant to integrate cost-effective health care options including e-health applications into a stepped care approach, as in the presented RCT. This fits right in with the importance that patient organizations, policy makers and researchers currently attach to e-health self-management tools.

Beside assessing overall efficacy of a stepped care approach targeting psychological distress in cancer patients, also insight will be obtained into possible determinants of the need for psychosocial care and success of a stepped care approach. These possible determinants include sociodemographic and disease and treatment related parameters, comorbidity, and quality of life.

Knowledge transfer of the results of the project on efficacy of stepped care targeting psychological distress in cancer patients into the scientific community includes submitting papers to (inter)national peer-reviewed journals, proceedings, and news letters and presenting papers at national and international conferences, both in early pilot stages and after conclusion of the project.

In case of positive results of this RCT on effectiveness, a second step aims at adaptation and maintenance of the stepped care approach to bring the evidence-based practice regarding improving distress in cancer patients into consistent and appropriate use in all oncological centers in the Netherlands. A sharing mechanism will be designed to facilitate adaptation and maintenance such as informing the Dutch Lung Cancer Study Group, Dutch Society of Pulmonologists (NVALT), Netherlands Society of Otorhinolaryngology and Cervico-Facial Surgery, Dutch Head and Neck Oncology Cooperative Group (NWHHT), Dutch Society of Psychosocial Oncology, oncological and psychiatric nursing societies and patient societies, to structure the results of this project into implementation projects in all oncological centers throughout The Netherlands. Guideline committees will be informed and advised to adapt the Nation-wide Guidelines on Laryngeal (2010, version 3.0), Lung (2004, version 1.0), Oral Cavity and Oropharyngeal (2004, version 1.4) and Hypopharyngeal (2010, version 2.0) Cancer [63-66].

The bottom line of the stepped care approach is healthier patients, more satisfied care providers, and cost savings by empowering both professionals and patients.

### Ethical considerations

This study is conducted in accordance with the Declaration of Helsinki and in accordance with local laws and regulations. Eligible patients are fully informed about the study and asked to participate. The patients receive a patient information sheet and have ample opportunity to ask questions and to consider the implications of the study before deciding to participate. Patients consent is noted on an informed consent form compliant with the local and ethical regulations. If during the study the patient for whatever reason no longer wishes to participate, the patient is allowed to withdraw his consent at any time. The study protocol has been approved by the Medical Ethical Committee of VU University Medical Center, Amsterdam, The Netherlands.

## Competing interests

The authors declare that they have no competing interests.

## Authors’ contributions

CRL, RB, AS, FS, PC, and IMVL contributed to the design of the study. The study is being coordinated by AMHK and IMVL. The present manuscript was drafted by AMHK en IMVL. All authors contributed to critical revision of the manuscript. All authors read and approved the final manuscript.

## Pre-publication history

The pre-publication history for this paper can be accessed here:

http://www.biomedcentral.com/1471-2407/12/173/prepub
